# Tracking Chronic Diseases via Mobile Health Applications: Which User Experience Aspects Are Key?

**DOI:** 10.3390/healthcare13243272

**Published:** 2025-12-12

**Authors:** Anouk S. Huberts, Preston Long, Ann-Kristin Porth, Liselotte Fierens, Nicholas C. Carney, Linetta Koppert, Alexandra Kautzky-Willer, Belle H. de Rooij, Tanja Stamm

**Affiliations:** 1 Department of Quality and Patientcare, Erasmus University Medical Center, 3015 GD Rotterdam, The Netherlands; 2 Institute for Outcomes Research, Center for Medical Data Science, Medical University of Vienna, 1090 Wien, Austria; 3 Department of Internal Medicine III, Division of Endocrinology and Metabolism, Medical University Vienna, 1090 Wien, Austriaalexandra.kautzky-willer@meduniwien.ac.at (A.K.-W.); 4 Department of Chronic Diseases and Metabolism, KU Leuven, 3000 Leuven, Belgium; 5 Pharma Personalised Healthcare, F. Hoffmann–La Roche AG, 4070 Basel, Switzerland; 6 Department of Surgical Oncology, Erasmus MC Cancer Institute, Erasmus University Medical Center, 3015 GD Rotterdam, The Netherlands; 7 Department of Research and Development, Netherlands Comprehensive Cancer Organization, 3501 DB Utrecht, The Netherlands; 8 Ludwig Boltzmann Institute for Arthritis and Rehabilitation, 1090 Vienna, Austria

**Keywords:** chronic disease, PROs, user experience, digital health

## Abstract

**Highlights:**

**What are the main findings?**

- Shared backbone features are essential: Patients across chronic disease groups prioritize integration with other apps/devices, seamless data sharing with healthcare teams, alerts, and reinforcement features.
- Customization is critical: While the backbone is shared, disease-specific and patient-specific interface customization (e.g., glucose monitoring, flare detection, side-effect tracking) is necessary for usability and engagement.

**What are the implications of the main findings?**

- Unified, transdiagnostic apps are preferable: A single app that serves multiple diseases can reduce patient burden, streamline data collection, and improve engagement for patients with comorbidities.
- Enhanced UX can improve healthcare outcomes: Designing apps with tailored, trustworthy, and user-centered features may not only increase participation but also potentially improve patient health outcomes, highlighting the importance of UX in digital health interventions.

**Abstract:**

**Background:** A key barrier to realizing the full potential and long-term collection of patient-reported outcomes (PROs) is the limited understanding of user experience (UX) factors that influence sustained patient engagement with digital PRO tools. Most existing research focuses on disease-specific or country-specific solutions, leaving a gap in identifying shared UX determinants that could inform scalable, cross-disease European digital health frameworks. This fragmentation hinders interoperability and increases development costs by requiring separate tools for each context. This case study aims to address this gap by identifying key UX features that optimize PRO collection across diverse chronic conditions in Europe within the Health Outcomes Observatory project, enhancing continuous (primary use) and large-scale (secondary use) data collection. **Objective:** This study aimed to identify and analyze key UX factors that support adoption and sustained use of PRO collection tools among patients with chronic diseases across multiple European countries. **Methods:** Patient focus groups were conducted in four chronic disease areas: cancer, inflammatory bowel disease (IBD), and diabetes (type I and II) across six European countries. Participants were recruited purposively through national patient advisory boards to ensure diversity in age, gender, and disease type. Sessions were moderated by trained qualitative researchers following a standardized guide, and discussions were transcribed verbatim and coded in researcher pairs to ensure intercoder reliability through iterative consensus. A modified thematic analysis, guided deductively by the UX Honeycomb model and inductively by emergent themes, was used to identify cross-disease UX determinants. **Results:** In total, 17 patients and patient representatives participated (76% female; 4 diabetes, 6 IBD and 7 cancer). We identified six core UX factors driving patient engagement for all disease groups: compatibility with other technologies, direct communication with the care team, personalization, ability to share data, the need for educational material and data protection were identified as key aspects of PRO technologies. However, the customizability of the app is crucial. Not all disease groups had the same needs, and participants specifically requested that the app provide information relevant to their own condition. Disease-specific needs, like T1D patients desiring glucose monitoring integration, were identified. IBD patients highlighted flare detection abilities and cancer patients especially sought side-effect comparisons. **Conclusions:** Our findings indicate that a unified yet customizable PRO platform can address shared UX needs across diseases, improving patient engagement and data quality. Incorporating features such as seamless data transfer, personalization, feedback, and strong privacy measures can foster trust and long-term adoption across European contexts. In addition to some disease-specific issues, most needs for the backbone of the app were shared among the disease areas. This shows that a shared app between diseases might be preferable and, in case of comorbidities, could ease self-management for patients. Last, to ensure full potential for every user and every disease, customization is crucial.

## 1. Introduction

Over recent years there has been growing interest in using patient-reported outcomes (PROs), which are direct self-reports of a person’s health condition without interpretation by a clinician or anyone else [1]. They can be measured and standardized by validated questionnaires, so-called patient-reported outcome measures (PROMs), most of which were initially developed for use in clinical trials to demonstrate the risks, benefits and safety of interventions. Furthermore, in real-world settings, PROMs can be used to highlight variation in outcomes, costs and processes at the service level, and trends and disparities at the population level (secondary use) [1,2]. At present, efforts are being made to employ them more and more at the individual level, that is, to use them in clinical interactions with healthcare providers (HCPs) to facilitate communication and support shared decision-making (primary use) [2,3].

To enable their systematic integration into healthcare, standardized outcome sets combining clinical and patient-reported indicators have been developed internationally [4,5]. Yet, large-scale implementations that simultaneously support clinical care and research remain rare. The Health Outcomes Observatory (H2O) project, funded by the Innovative Medicines Initiative (IMI), bridges this gap by creating a data governance and infrastructure system across Europe to allow for collecting patient outcomes and integrating them into healthcare decision-making to enable health systems to operate more sustainably and support individual healthcare delivery [6]. Its initial pilot focuses on diabetes (type 1 and 2), inflammatory bowel disease (IBD), and oncology (lung, metastatic breast, and hematologic cancers), aiming to expand to other conditions and countries.

The success of such initiatives depends heavily on digital tools that make PROs accessible, interpretable, and sustainable through electronic means (ePROMs) [7,8,9,10,11]. Integrating digitalized PROs into disease registries can maximize their value to key stakeholders, such as patients, healthcare providers, policy makers and researchers [12]. Mobile health (mHealth) applications represent a promising vehicle for ePROM collection, offering real-time data entry, enhanced engagement, and potential behavior change support. By offering a user-friendly and accessible platform, mobile apps encourage patients to regularly provide feedback, leading to more accurate and continuous monitoring of their health [7,13,14]. For individuals with chronic conditions who interact with digital tools repeatedly and over long durations, sustained engagement depends on an optimized user experience (UX). UX has been described as the user’s subjected, situated, complex and dynamic experience and different models have been proposed to apply the UX perspective to application design [15,16,17]. An application can be considered usable when it enables specific users to reach context-specific goals in an effective and satisfying manner [17]. Good UX will unlock the data’s potential, increase user retention, and help deliver the benefits of ePROMs [15,16,18,19].

Yet, existing UX research in healthcare remains fragmented, largely disease-specific, nationally bounded, and based on small or homogeneous samples [7,20,21,22,23,24,25,26,27,28,29]. Little is known about cross-disease and cross-country UX factors that sustain long-term engagement with digital PRO tools, limiting scalability across conditions and healthcare systems. Furthermore, while multiple pathologies should be considered within one framework, patients should also be consulted from initial conception of the research through to resulting satisfaction and acceptance [30,31,32]. Few studies involve patients from the initial exploration of the design and continue to do so throughout the co-design process [26,27,28].

Therefore, this study aims to identify cross-disease UX features that support effective and sustainable PRO collection across chronic conditions and European contexts, contributing to the design of scalable, patient-centered digital health infrastructures.

## 2. Materials and Methods

We conducted semi-structured focus group interviews with people living with diabetes, cancer, or IBD and patient representatives to explore their experiences with and expectations towards digital health solutions for collecting PROs. By including participants from different countries, this study tries to enhance the cultural, linguistic, and healthcare system diversity between countries, making digital tools more adaptable to various user needs. It ensures that the tool is usable across different regions, addresses varying health behaviors and is compatible with diverse healthcare settings, ultimately increasing its generalizability and global relevance. The interviews were held between September and December 2021 (By A.S.H., A.K.P., P.L., B.d.R., L.F., N.C.C.). The questions were derived through an expert focus group comprising researchers and clinicians, including representatives from each disease area. We followed the consolidated criteria for reporting qualitative research (COREQ) checklist to ensure high-quality reporting of the study results [33].

### 2.1. Participants and Data Collection

Participants were recruited from the H2O patient advisory boards (PABs) for diabetes, IBD and cancer, which includes patients and patient representatives across Europe. Three online focus groups were conducted—one per disease area (diabetes, IBD, and cancer)—each comprising 4–7 participants from multiple European countries. To be eligible for the study, participants had to (1) be diagnosed with either Type 1 or Type 2 diabetes (T1D or T2D), lung or breast cancer or a hematologic malignancy, or IBD, or be a patient advocate, representing one of these patient populations; (2) be older than 18 years and able to provide consent; and (3) be proficient in English. The group consisted of patients with high health literacy and higher socio-economic status.

We used the same semi-structured interview guide for all focus groups to enable focused discussions on aligned topics and comparability across conditions and geographic locations (Box 1). It consisted of four main open-ended questions and suggested optional probing questions, allowing for flexibility in the conversation. The focus groups were held in English via online videoconference and lasted between 60 and 90 min. There were no strict times set for breaks, but those were included if needed. Although all discussions were held in English, moderators rephrased and clarified expressions to accommodate participants with varying fluency levels. Each interview was facilitated by experienced clinical researchers, audio-recorded with the consent of all participants and then transcribed using Amberscript software (https://www.amberscript.com, Amberscript Global B.V., Amsterdam, The Netherlands). To ensure interpretive validity, summaries of key discussion points were shared with participants (member checking) for confirmation and correction.

Box 1 **Interview guide questions on expectations and needs of people living with chronic conditions in terms of digital health solutions.**

Guide for patients focus group interviews on digital health solutions

- What experiences do you have with health tracking apps? What did you like, what did you not like and what did you feel was missing?
- For you, what purpose should a health tracking app fulfil regarding diabetes, cancer or IBD care and what functions would be needed for this in your opinion?
  Possible probe questions related to functions:
  ∘ directly linking other devices or apps to disease-specific application/devices (e.g., diabetes tracker).
  ∘ reminder function (e.g., measuring blood glucose, taking medication).
  ∘ notifications concerning the course of the disease, e.g., blood glucose trends.
  ∘ the possibility to remotely be connected with the HCP via the app.
  ∘ a social network function and educational content integrated into the app.
- How would you like to see the data that you collected displayed and reported back to you?
- How much time are you willing to spend on entering data into a health tracking app? How many questions would you be willing to answer—and in your opinion, how often would be appropriate?

### 2.2. Data Analysis

We followed a modified thematic analysis with elements of meaning condensation [34]. After initial screening of the material, trained researchers representing each disease area (AH, AKP, BR, LF, NCC) in the study team coded the interviews in pairs. Meaning units were identified as coherent statements reflecting a single idea. An evolving codebook was maintained, which was reconciled in cross-disease meetings to ensure consistency and to assess intercoder agreement through consensus discussion. Within this series of meetings, discrepancies in the coding scheme were resolved. At this stage, the researchers also identified potential themes. Relationships between the initial codes were identified, resulting in a more structured list of codes, organized in candidate themes. Subsequently, the themes were color-coded according to the initial researchers and a human factors specialist (PL). Codes were assigned to a disease area so that commonalities and differences regarding the technological needs of people living with different chronic conditions could be recognized. The themes represented specific features which participants felt were important for good UX.

Following inductive coding, themes were deductively aligned with the UX Honeycomb framework [35,36]. This dual (inductive–deductive) approach allowed participant-driven insights to be contextualized within established UX dimensions (usefulness, usability, desirability, findability, accessibility, credibility, and value) [37,38]. Five trained researchers independently cross-referenced the derived themes with the elements. Any discrepancies were resolved during various meetings. Each theme was linked to 1–3 UX elements based on unanimous agreement among the researchers.

### 2.3. Ethical Requirements

Ethical approval was obtained from the Medical University of Vienna (EK1472/2021). The Dutch Medical Research Act did not apply to this study, which was confirmed by the local Medical Ethics Review Committee (MEC-2021-0776). As these two studies led the work, no other ethics were required despite patients from other countries participating.

## 3. Results

In total, 17 people from six European countries were included in the three focus groups. In the cancer group, seven patients participated in a focus group and represented breast cancer (n = 1), lung cancer (n = 2) and hematologic cancer types (n = 1) or represented cancer patients in general (n = 3). In the IBD group, six patients (50% Crohn’s disease) participated, and in the diabetes group, four patients (50% Type 1) participated. The following countries were represented in the aforementioned groups: Belgium, Germany, Austria, Spain, Greece and Hungary. Detailed demographic information of the participants is displayed in Table 1.

The coding resulted in 25 subthemes, mainly relating to self-management support, establishing contact with healthcare providers and peers, and receiving information. They represent important features which need to be considered for good UX according to the participants’ testimonies. The themes and how these were mapped to the UX Honeycomb are shown in Supplementary Figure S1 and Supplementary Table S2.

### 3.1. Top Features of an Ideal Digital Solution for People with a Chronic Condition

**Figure 1:**
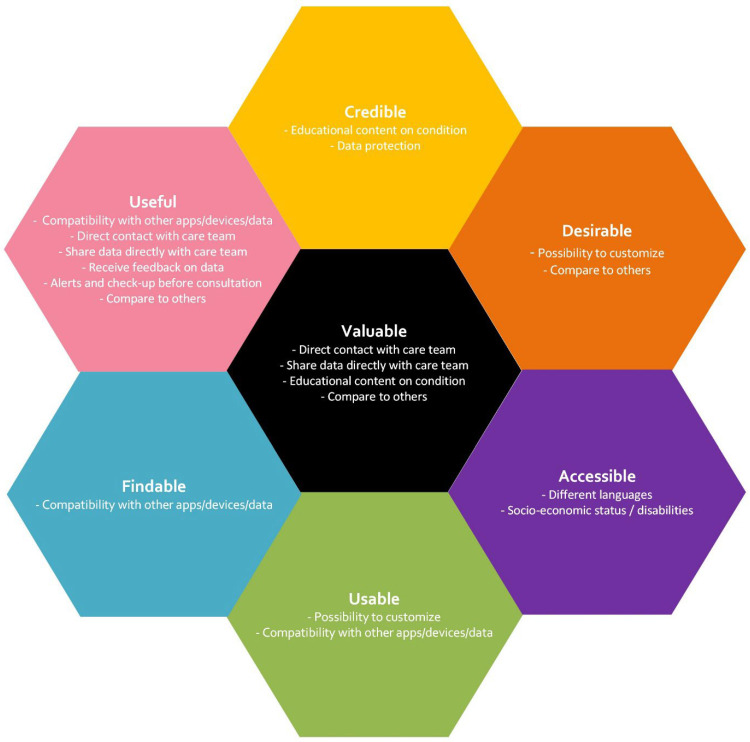
Most important features for a PRO collection tool for different chronic diseases. Themes are randomly ordered, irrespective of perceived importance or disease groups. Themes mapped to one or more categories are presented in all categories.

During our analysis, eleven features stood out as being the most important to be included in a digital health application. A feature was deemed important if it was mentioned by two or more disease groups or perceived as particularly significant by one group, meaning it was independently referenced multiple times within that group. They are illustrated using the UX Honeycomb in Figure 1 and described in more detail below. This list is not exhaustive and other features also need consideration when developing technical solutions, but they were perceived as less important by our participants. The quotes presented are intended to capture a core feature and reflect multiple comparable inputs.

#### 3.1.1. Possibility to Customize Apps (Usable, Desirable)

All disease groups noted that a digital health application needed to be customizable, i.e., it was important to them that they could select the data fields and features displayed according to what they considered important. They explained that customization would render an app easy and ready to use as not everyone wanted to see all available data and information all the time. One participant explained that too much data could cause extra stress, because it would enable them to check every moment of the day how their condition was developing. However, others stated that keeping track of comprehensive data improved their quality of life. To avoid negative emotions regarding the app (desirability), customization is key.

“*I think if you list all the options, all the features and that you can choose that you only want some of these features available in your tool. That would be great.*”

*(IBD)*

#### 3.1.2. Compatibility with Other Apps/Devices and Data (Useful, Usable, Findable)

Participants of all disease groups mentioned that the combination of comprehensive functions would add to a new digital system’s usefulness, usability, and findability. They wanted it to combine (all) benefits of existing systems and most importantly automatically integrate data from different sources such as apps, devices and systems used by them and their care team. They pointed out that a vast number of health apps for many different purposes already exist, and that they perceived many of them as time-consuming. An app that could replace the use of several apps would therefore be highly useful. The backbone of a good working app could facilitate this. Nonetheless, the disease groups did mention different examples of data integration, stressing again the need for customization of the user-facing interface.

“*So I would be very likely to use it if I had all the information about myself in there my clinical information, my magazines, the information about my disease, what to do if this happened or other things happen. I mean, and maybe an email or a contact. And if through a direct contact with an answer to the health care providers, I think that would be very useful.*”

*(Cancer)*

People with diabetes explained that comprehensive data integration would be useful for their self-management. They saw benefit in integrating disease-related functions, such as blood glucose monitoring, with features to facilitate self-management, e.g., a calculator function, sufficient nutritional support, and exercise.

“*You might miss it [a hypo] and you might go running, you find yourself in very high blood sugar levels or vice versa. You might double it and go for an exercise and you find yourself in a hypo in a place where you don’t want to be with the hypo. So the thing is that that [integrating data] was a very good element for my management*.”

*(Diabetes)*

Furthermore, an IBD patient mentioned that care in the Spanish health system is very fragmented and that it is hard to transfer data between different HCPs. This supports the fact that a technical solution needs to overcome these obstacles by enabling high compatibility and data transfers between hospitals and devices.

“*So I live in one region of Spain and my gastroenterologist is in another region of Spain, and the Spanish health care system cannot facilitate our communication between the two different parts of the country. So I’m just wondering how [the] H2O [project] is going to be able to overcome these obstacles [by using an app] that I find now in my day to day just within the same country?*”

*(IBD)*

#### 3.1.3. Direct Contact with Care Team (Useful, Valuable)

Direct communication with the care team was perceived as important by participants of all disease groups. They expressed the wish that a digital solution would enable direct contact with the appropriate HCP, depending on the problem they needed support with. Such a feature can add value for both patients—who can be considered the customers in the UX framework—HCPs and the app supplier, which ultimately defines the value of a solution. In this way, the HCP could collect the required information before the start of the consultation, saving time and resources, which is valuable in the clinical environment.

“*…if you have a question on Wednesday, then she can talk to you on Tuesday, the week after. So (…) I don’t like that. I really want a doctor who is available pretty quick.*”

*(Cancer)*

“*… if you feel like you need an appointment because a flare is coming up, that you can have immediate assistance. It would be great if that would be possible.*”

*(IBD)*

#### 3.1.4. Share Data Directly with Care Team (Useful, Valuable)

Transferring data, both PROs and self-reported outcomes, entered into the app by patients directly to HCPs was seen as useful and valuable as it can support improving the quality of consultations or even replacing (face-to-face) consultations. Moreover, it can save time and better inform the HCP, especially in the care of patients with type 1 diabetes as glucose monitoring produces a lot of data.

“*When I got a device that was capable of transferring data into a database I could share with my doctor. That was again, something very helpful for me to go back and see if something happened with the doctor. What was the cause of it? What should I do? What should I not do in the future, just to avoid delicate situations?*”

*(Diabetes)*

“*… to shorten the conversation because the clinician doesn’t have to ask all the questions again. They can have a look at the results of the app (…) and then we could step into a constructive conversation about the problems I currently have to deal with and how to solve them.*”

*(IBD)*

Moreover, the possibility of direct data transfer gave participants a feeling of security as possible serious side effects can be captured by the care team before even noticing themselves.

“*And it could be a very positive to have, I don’t know, quickly monitoring of the patient that instead of calling the nurse or calling the doctor, you can receive a call from the hospital.*”

*(Cancer)*

#### 3.1.5. Socio-Economic Status/Disabilities (Accessible)

All disease groups were concerned about the universal accessibility of the technology, including accessibility for patients with limited access to the internet, e.g., due to a person’s socio-economic status, technical affinity or other individual factors and life circumstances. It was also mentioned that the needs of people with other disabilities should be considered. On the other hand, many people nowadays have access to a smart phone. A mobile app was therefore considered as a good solution.

“*Maybe think of accessibility for people with disabilities. So you that that you have people who can’t see very well or not, that is something that you have to keep in mind because you will have those patients as well who have maybe other problems.*”

*(IBD)*

“*Maybe older patients can have some problems, but most of the cases most people have seen as we have smartphones, everybody, we are used to use apps. I think that being a mobile app that can be used daily by the patients are pretty useful.*”

*(Cancer)*

#### 3.1.6. Different Languages (Accessible)

Availability in multiple languages was considered important for accessibility, including disease-specific information and studies.

“*There is a big barrier because most of the information is in English. (…) So this is the biggest barrier which should be considered in any case.*”

*(Cancer)*

#### 3.1.7. Educational Content on Condition (Credible, Valuable)

The desire for credible information about their condition was mentioned by many participants irrespective of the condition they were living with. In this context, the participants mentioned newsletters, information on where to go with disease-related troubles, informative content about the condition and information about welfare and financial support. Providing this information alongside collected health status data would add to an app’s credibility and was considered relevant due to the abundance of information available online about which participants said they were unsure of what to believe. Moreover, in some countries, HCPs, as the primary source of credible information, are not always within reach. Provision of credible information can therefore withhold people from going to the hospital if not needed, allowing resources and time to be saved. Information about who is responsible for the app and possible problems was only mentioned by participants of the cancer focus group; however, it would also enhance the credibility. A difficulty with this factor is filtering and providing credible information from the vast amount available that is most relevant to patients. Such material may also require updating overtime to ensure that any information provided remains up to date.

“ *Like you have a leaflet for the medication. You know, it should not be like a computer where you have thousand millions of knowledge, which can be downloaded. It should be tailored to your problems, your worries. (…) As a personalized thing where you can find the information which are to be available, which are to be applied only to your specific disease.*”

*(Cancer)*

“*So the app or the application or whatever is very important just for the Eastern Europe because they’re there that in some countries in Eastern Europe, where the doctor is not available, if you have a problem that you can get the information instead of going to the hospital.*”

*(Cancer)*

#### 3.1.8. Receive Feedback on Data (Useful)

To ensure user retention, receiving feedback on the data was important to all disease groups. They stated that if patients did not receive feedback, digital data collection would not feel useful for them and their motivation would drop fast.

“*Otherwise [if no feedback is received] I do not see why to feel and spent time in filling in something.*”

*(IBD)*

“*So this [answering PROMs] could be good, but you need to know that you’ll receive feedback. Often times you fill in the questionnaire for someone but you don’t know what they are going to do with this data and they don’t give you feedback. But if you have an illness, I think and you’re interested and want to put this data in the questionnaire, but you need feedback, a continuous feedback and improvement of your health thanks to the time you’re spending with the questionnaire.*”

*(Diabetes)*

#### 3.1.9. Alerts and Check-Up Before Consultation (Useful, Valuable)

Different types of notifications were considered useful and valuable features of an app in all groups. Reminders to take medication, as well as alerts and check lists for new consultations could contribute to improving treatment adherence, reduce no-shows, and improve the consistency of consultations and therefore add value to the whole care chain.

“*The idea of being able to track appointments, schedule appointments and all of that would be amazing.*”

*(IBD)*

#### 3.1.10. Compare to Others (Useful, Desirable, Valuable)

Most participants found it motivating to be able to share information with peers (e.g., via a chat function) and compare their data with others (or to aggregated data). Irrespective of the condition they were living with, they mentioned that this would give them a feeling of self-control, which was an important feature rendering an app desirable for a lot of patients. Such a feature was also considered valuable, as it would allow them to learn how to improve their symptoms based on data provided by others.

“*And what I am also missing and I would like to see is how do I compare with other people? And so I have, you know, sort of a score, whether I am doing well, I’m sleeping well, I mean, eating well, etc.*”

*(Cancer)*

However, also for this feature customization, i.e., the ability to choose if and with whom someone wants to compare themselves/their data is important. Specifically, participants with diabetes voiced scepticism in relation to this feature and its desirability, as they believe every person is different and has a very individual diabetes trajectory.

“*I would not be motivated by comparing myself with others. Simply because I have seen over the years: Many people are very different, and different actions have very different effects on the sugar treatment.*”

*(Diabetes)*

#### 3.1.11. Data Protection (Credible)

Participants within the cancer and diabetes groups mentioned that data protection was very important to them. They emphasized that if good data protection was in place and if this was communicated well, it would increase the app’s credibility.

“*What happens with my data? Again, that’s something we need to be very careful on how to keep anonymity*.”

*(Cancer)*

## 4. Discussion

This study investigated UX preferences for digital health applications for collecting PROs for chronic disease management. Patients with chronic diseases must frequently use their respective health applications over extended periods, making a positive user experience essential. A positive experience also supports patients ‘sense of autonomy, attitude and competence, which are key psychological drivers in the Self-Determination Theory and Technology Acceptance Model (TAM). Additionally, using these apps can be more burdensome for individuals with chronic conditions compared to those who are healthier, highlighting the need for tailored support. The findings contribute directly to the Health Outcomes Observatory (H2O) initiative by identifying empirically grounded design priorities that can inform scalable, cross-disease ePRO infrastructures in Europe.

This study shows that, according to patients, essential backbone features of a well-designed app are shared across different disease groups, which included compatibility with other applications and devices, integration of various data sources, as well as the seamless sharing of data with the patient’s healthcare team. This indicates that patients are reluctant to adopt yet another standalone (PRO collection) app, in particular when their reported data cannot easily be integrated and used with other health data. Indeed, others have reported that patients are reluctant to collect and share PRO data if they do not experience a direct benefit for their care from this [39]. Patients’ reluctance to use yet another standalone app reflects not just frustration but a deeper need for system coherence. A seamless data transfer would not only save time and resources, but may also enhance communication with an between HCPs and therefore the primary use [8,12,40,41]. When combined with app compatibility, these features can significantly reduce the time patients need to spend switching between different apps and entering data [12,42]. This is an important step for future healthcare, as most apps are now still developed for single purposes or separate diseases (see Supplementary Table S2). The collection of data of people with different diseases or comorbidities by one app, can improve data quality for both primary and secondary use. Moreover, patients with comorbidities do not have to answer questions regarding case mix variables, or general PROs multiple times. However, differences in the user-facing interface were highlighted, making customizability a key aspect of app design.

Subsequently, a new app was perceived usable when multiple factors of existing apps can be combined with new features such as direct contact with the care team, alerts and check-ups, and personalization options. This is in line with earlier research, stating that near-daily use was caused by the ability of patients to self-manage and patient-centered care delivery and not purely due to ePRO collection [7,43]. Moreover, reinforcement and communication are design features of mobile health interventions and behavior change techniques that can improve the effectiveness of the app [44,45]. H2O believes that reinforcement of patients can be promoted via patient dashboards on which they receive feedback on their own data. Personalization, reinforcement, and communication can improve user participation and promote the effectiveness of interventions [46]. While some of these findings align with disease-specific features observed in other studies, our research demonstrates that these desired features are shared across different diseases and European countries (see Supplementary Table S2).

Credibility was another significantly represented domain. According to our findings, patients found strong data protection and trustworthy educational information important factors to increase the credibility of the app. Data protection is especially important in healthcare, where sensitive data should be stringently safeguarded against misuse [10]. Moreover, IBD, diabetes and cancer patients have expressed their wishes for good, concise educational information in earlier studies [43,47,48,49]. This factor seems increasingly important in the current age of misinformation and the rising complexity of healthcare [50].

The features mentioned above are essential for people living with one of the three diseases included in this study. These features form the backbone of the app, supporting the H2O concept of creating a unified framework for data collection. Instead of developing separate apps for each disease, which would need to communicate with EHRs in different ways, a single backbone could streamline data collection. However, the emphasis on app compatibility and EHR integration also reflects a tension between individual usability and systemic constraints. UX design must account for institutional interoperability barriers as part of the user journey, balancing simplicity for patients with compliance and standardization requirements for providers. Yet, achieving this level of integration remains complex in practice, as digital infrastructures and data governance regulations differ widely across European healthcare systems. Variations in technical standards, privacy legislation, and EHR vendor ecosystems can significantly hinder seamless data exchange. Designing an application that accommodates these structural and regulatory differences is therefore essential to ensure both scalability and patient trust in cross-border ePRO collection.

The identified need for customizable, disease-specific interfaces highlights how personalization shapes both perceived relevance and engagement. This aligns with behavior change theories emphasizing tailoring and the adaptation of interventions to users’ specific contexts to enhance motivation and adherence. For example, the desire for glucose monitoring among T1D patients or flare detection in IBD demonstrates that perceived usefulness (TAM) is highly context-dependent. At the same time, the challenge lies in maintaining a coherent core framework while allowing flexibility in surface-level customization. This trade-off between standardization and personalization represents a key design tension in cross-disease digital health solutions.

Despite some disease-specific nuances, shared chronicity led to core commonalities in the backbone of the app. The use of multiple focus groups has been found to achieve approximately 80% saturation with sample sizes of 9–17 [51,52]. Furthermore, while all participants reside within the EU, each of the six countries included has a distinct culture, particularly between the eastern and western regions. Thus, our multi-disease, international focus group strengthens the generalizability of these findings to other chronic disease areas. Moreover, as comorbidity in patients will rise, it is of importance that an app can serve multiple diseases. Transdiagnostic apps can increase patient engagement and treatment efficacy by reducing the commitment needed to interact with multiple apps for comorbid disorders [53].

The study’s sample consisted largely of expert patients and patient advocates and a majority were female. It was also a sample of convenience of patients participating in the H2O project. While this provided in-depth insights, it may have led to an overrepresentation of digitally literate perspectives. Moreover, gender differences might also influence the UX. Future studies should therefore include more diverse populations, including non-English speakers, individuals with lower digital literacy or socio-economic barriers, to assess whether the identified UX priorities across subgroups improve the generalizability further. Furthermore, the focus groups held no breaks to mitigate fatigue, and the summaries generated were not confirmed with the patients, both of which may have compromised the data quality. On the other hand, app development typically proceeds through small focus groups, since it involves detailed discussions with patients. We therefore believe that the result of this study provides proof that points to the development of a single, unified application.

Further research should therefore focus on app usage among patients with comorbidities. Although we compared the three disease groups, little is known about the willingness of patients with comorbidities to engage with an app. It should also focus on patients with different social economic status. Moreover, it should explore transferability to acute disease patients, evaluate previously existing apps for their appropriateness of use with a chronic disease population using the factors noted here, and most vitally, study the impact of improved user experiences on health outcomes. A formal impact study could offer the first empirical evidence for the benefits of using a well-designed health application directly on the user’s (patients) health outcomes. The direct establishment of improved user experiences (e.g., positive user experiences in an IBD app predictive of decreased intestinal discomfort) with improved health outcomes would invigorate UX healthcare research and increase the perceived resulting value of considerate designs for all relevant stakeholders.

Lastly, artificial intelligence (AI) offers promising opportunities to enhance PRO tools by addressing key challenges in usability, engagement, and data integration. AI-driven personalization could tailor interfaces and feedback to individual needs, improving long-term adherence across diverse patient groups. Machine learning and natural language processing can streamline data integration and enable intuitive, conversational reporting while supporting automated qualitative analysis. Privacy-preserving approaches, such as federated learning, can strengthen trust and data security. By identifying shared engagement patterns across diseases and contexts, AI can also inform scalable, interoperable frameworks for PRO collection. Collectively, these applications could transform PRO tools into adaptive, intelligent systems that promote sustained, patient-centered engagement.

## 5. Conclusions

In conclusion, this study highlights that effective PRO-collection applications for chronic disease management must balance a shared, interoperable backbone with flexible, disease-specific customization. Key design principles—interoperability, real-time feedback, credible data handling, and user-centered personalization—represent not isolated preferences but interconnected determinants of trust and sustained engagement.

Transdiagnostic digital tools that incorporate these UX dimensions could reduce patient burden, strengthen clinical integration, and ultimately improve the quality and continuity of outcome data across Europe. Continued collaborative design and empirical evaluation are needed to translate these insights into scalable, equitable, and future-proof health technologies.

## Figures and Tables

**Table 1:** Focus group participant demographics.

	Total (n = 17)	Diabetes (n = 4)	IBD (n = 6)	Cancer (n = 7)
	N (%)	N (%)	N (%)	N (%)
**Diagnosis**				
** ** **Type 1 diabetes**	2 (12)	2 (50)	-	-
** ** **Type 2 diabetes**	1 (6)	1 (25)	-	-
** ** **Crohn’s disease**	3 (18)	-	3 (50)	-
Ulcerative colitis	1 (6)	-	1 (17)	-
Breast cancer	1 (6)	-	-	1 (14)
Lung cancer	2 (12)	-	-	2 (29)
Hematologic cancer	1 (6)	-	-	1 (14)
Patient advocate	6 (35)	1 (25)	2 (33)	3 (43)
**Gender**				
Male	4 (24)	1 (25)	-	3 (43)
Female	13 (76)	3 (75)	6 (100)	4 (57)
**Age group**				
20–29	1 (6)	-	1 (17)	-
30–39	3 (18)	1 (25)	2 (33)	-
40–49	4 (24)	1 (25)	2 (33)	1 (14)
50–59	3 (18)	1 (25)	-	2 (29)
60–69	3 (18)	1 (25)	1 (17)	1 (14)
70–79	2 (12)	-	-	2 (29)
Missing	1 (6)	-	-	1 (14)

IBD = inflammatory bowel disease.

## Data Availability

The original contributions as quotations are presented in this study and are included in the article/Supplementary Material. Further inquiries can be directed to the corresponding author.

## Supplementary Materials

The following supporting information can be downloaded at https://www.mdpi.com/article/10.3390/healthcare13243272/s1, Table S1: Subthemes mentioned by the disease groups with accompanying quotes. Table S2: Literature on apps for PRO collection and UX studies. Figure S1: Themes for the focus groups mapped to the Honeycomb model.

## Abbreviations

The following abbreviations are used in this manuscript:

CGM	Continuous glucose monitoring
ePROMs	Electronically available Patient-Reported Outcome Measure
H2O	Health Outcomes Observatory
HCP	Healthcare professional
IBD	Inflammatory Bowel Disease
IMI	Innovative medicines Initiative
PRO	Patient-reported Outcome
PROM	Patient-reported Outcome Measure
PAB	Patient advisory board
T1D	Type 1 diabetes
T2D	Type 2 diabetes
UX	User experience
