# Supplementary material for: Tracking Chronic Diseases via Mobile Health Applications: Which User Experience Aspects Are Key?
Source: Healthcare (Basel). 2025 Dec 12;13(24):3272. doi: 10.3390/healthcare13243272 (PMC12733319; doi:10.3390/healthcare13243272)
Supplement: Supplementary file 1 [file healthcare-13-03272-s001.zip › healthcare-3956455-supplementary.pdf]

Supplementary Table S1: Subthemes mentioned by the disease groups with accompanying quotes.

Subthemes	Diabetes	Cancer	IBD	UX category	Quotes
Compatibility with other apps/ devices and data	X	X	X	Useful, Usable, Findable	So I live in one region of Spain and my gastroenterologist is in another region of Spain, and the Spanish health care system cannot facilitate our communication between the two different parts of the country. So I'm just wondering how H2O is going to be able to overcome obstacles that I find now in my day to day just within the same country? (IBD)<br>-----------------------<br>So it would be I would be very likely to use it if I had all the information about myself in there my clinical information, my magazines, the information about my disease, what to do if this happened or this other thing happens. I mean, and maybe an email or a contact. And if through a direct contact with an answer to the health care providers, I think that would be very useful. (Cancer)<br>-----------------------<br>It is nice if they record automatically and let you see those records over a certain amount of time so that you can find patterns in your data and kind of see if there are any triggers (like nutrition or exercise) that led to certain events. (Diabetes)
Direct contact with care team	X	X	X	Useful, Valuable	An IBD nurse is probably maybe a solution, if you feel like flares coming up or something like that, that you can have immediate assistance that could be great if that would be possible. That would be good if you could receive a quick, quick assistance medical assistance. (IBD)<br>-----------------------<br>I think that it's important from my care team to be an active participant in the app. But to be honest, I don't think that the doctors in Greece have the time to participate in interactive and have an active role in. They are because they are a little a number (IBD).<br>-----------------------<br>And if through a direct contact with an answer to the health care providers, I think that would be very useful. (Cancer)<br>-----------------------<br>I would say monthly management, looking back at data, filling it in and whenever necessary, communicating with my physician just to show that the haemoglobin values are actually reflected in the glucose excursion. (Diabetes)
Share data directly with care team	X	X	X	Useful, Valuable	"When I got a meter that was capable of transferring data into a database I could share with my doctor. That was again, something very helpful for me to go back and see if something happened with the doctor. What was the cause of it? What should I do? What should I not do in the future, just to avoid delicate situations?" (Diabetes)<br>-----------------------<br>Also it is nice to be able to share your data, especially with a doctor who can give you feedback on the data. And this feedback is the biggest motivation for you to enter the data (Diabetes)<br>-----------------------

					And it could be a very positive to have, I don't know, quickly monitoring of the patient that instead of calling the nurse or calling the doctor, you can receive a call from the hospital. (Cancer)
Different languages	X	X	X	Usable, Accessible	There is a big barrier because most of the information is in English. (...) So this is the biggest barrier which should be considered in any case. (Cancer)
Possibility to customize	X	X	X	Usable, Desirable	I think if you list all the options, all the features and that you can choose that you only want these features available in your tool. That would be great (IBD).<br>-----------------------<br>I think it's important to add some personalized goals because this reflects quality of your life when you're speaking with your IBD gastroenterologist or nurse and he can see you or she can see that you reached your personalized lives. Well, this yeah, this can be evidence why the person is feeling good or bad, or I think it would be good to add this to the app.(IBD)<br>-----------------------<br>It's very difficult because every disease have other features, and within every disease, every not every person is the same. So you, you need to personalize it. (Cancer).
Educational content on condition	X	X	X	Credible, Valuable	In our country, it will be very important to know where are the centres are, because it happens very frequently that my doctor is not anymore working for the hospital or the outpatient clinic where I used to go. So that would be also important to know where can I find a good center where I can go for the next time. Or if I have any troubles, any problems I can, I can go there. (IBD)<br>-----------------------<br>Like you have a leaflet for the medication. You know, it should not be like a computer where you have a thousand millions of knowledge, which can be downloaded. It should be tailored to your problems, your worries. (…) As a personalized thing where you can find the information which are to be available, which are to be applied only to your specific disease (Cancer).<br>-----------------------<br>So the the app or the application or whatever is very important just for the Eastern Europe because they're there that in some countries in Eastern Europe, where the doctor is not available, if you have a problem that you can get the information instead of going to the hospital (Cancer)<br>-----------------------<br>How soon after physical activity does my blood sugar go down? Maybe. And be able to tweak better, you know, like basal rates and things before actually doing an activity? That's stuff I generally work on with my doctor, but I don't think often enough. (Diabetes)
Socio-economic status / disabilities	X	X	X	Accessible	Maybe think of accessibility for people with disabilities. So you that that you have people who can't see very well or not, that that is something that you have to keep in mind because you will have those patients as well who have maybe other problems. (IBD)
Receive feedback on data	X	X	X	Useful	to shorten the conversation because he doesn't have to ask all the silly questions once he gets once again, he has a look at the of you knows everything, and then we really could step into a constructive conversation about the problems I now have to deal with and how to solve them (IBD).<br>-----------------------<br>Otherwise [if no feedback is received] I do not see why to feel and spent time in filling in something (IBD)<br>-----------------------

					"So this (answering PROMs) could be good, but you need to know that you'll receive feedback. (Often times) you fill in the questionnaire for someone but you don't know what they are going to do with this data and they don't give you feedback. But if you have an illness, I think and you're interested and want to put this data in the questionnaire, but you need feedback, a continuous feedback and improvement of your health thanks to the time you're spending with the questionnaire." (Diabetes)
Alerts and check-up before consultation	X	X	X	Useful, Valuable	The idea of being able to track appointments, schedule appointments and all of that would be amazing (IBD)<br>-----------------------<br>Alerts when you have a medical consultation or when you have, for instance, I get an injection every six months, so now I have an alert on my phone myself. But to have this alert would be helpful. Also, this type of alerts if you need to go to do an analysis, for instance. (Cancer)
Compare to others	X	X		Useful, Desirable, Valuable	"In social media, I would explicitly advise against comparing yourself with others, because you can only ever find the perfect curves [...] it's more depressing than motivating. [...] (But) If you have benchmarks and you can compare yourself, then I think that's great and motivating in any case." (Diabetes)<br>-----------------------<br>"I see their (other people with T1D) data. So it's like having a game, like wanting to be better than the other one, but also learn about what the others do, what exercise and learn, how exercise it affects their blood glucose, to feel that there are people like you, know that it's OK, they do this and they have more or less the same level of glucose or EPO or whatever they want to share." (Diabetes)<br>-----------------------<br>"I would not be motivated by comparing myself with others. Simply because I have seen over the years: Many people are very different, and different actions have very different effects on the sugar treatment." (Diabetes)<br>-----------------------<br>one of the things for me that I think I would personally like to see is access to data about other patients in the context of like, I'm thinking about myself, like I'm on chronic pain, on the same meds for years and years, and I would love to know other patients with the same type of diagnosis. The same, the same treatments like. Do they ever change, should they ever go on different meds, it is to have that kind of access to information (IBD)<br>-----------------------<br>And what I also missing and I would like to see is how do I compare with other people? And so I have, you know, sort of a of a of a score, whether I am doing well, I'm sleeping well, I mean, eating well, etc.. (Cancer)
Data protection	X	X		Credible	What happens with my data? Again, that's something we need to be very careful on how to keep anonymity. (Cancer)
Ability to collect clinical values and PROs	X		X	Useful	So what I'm talking about is that the device which is going to be, you know, implemented may be, you know, should include this monitoring [clinical data], which should be like a standard for everybody, no matter what your condition is (Cancer).<br>-----------------------

			They all know they have cancer and what they want to do is improve the quality of life. (…) You can inform our healthcare providers about your supply, side effects and how you feel. (Cancer)<br>-----------------------<br>I think that a device should include, in addition to the monitoring system of the vital things such as heart cholesterol, whenever this may be, is, you know, or some other, some other functions. (Cancer)
Forum / chat function with peers	X X	Desirable	I think it would be nice to have (something) like ... I know there is diabetes online communities: I used to participate in forums and stuff like that, but maybe something more (...) intuitive where you can jump on and chat to someone. I think that's especially important after someone is first diagnosed. I know it was for me to be able to connect with people around the world and talk to them about their experiences, just not necessarily, obviously, for medical advice, but just to be able to feel like you weren't alone in it. So that would be an interesting feature. I think (Diabetes)<br>-----------------------<br>So support from my peers is important. Just to know that I have less energy is totally normal in certain phases of your disease or other complaints (Cancer)
Monitoring disease	X	Useful	If any social event happens, I can track it back and see if it makes sense or if I can find the origin or the cause for that particular unpleasant event.<br>(Diabetes)
Monitoring medication	X	Useful, Valuable	I loved one piece of equipment. It was a insuline pen with a memory. [...] if you do something across your day every day for a good number of years, you get to a point when you cannot necessarily remember: 'did I do it or was it only my impression that I did?' [...] it is very good if you cannot overdo it with your medication by yourself. (Diabetes)
Automatic data recording	X	Useful, valuable	It is nice if they record automatically and let you see those records over a certain amount of time so that you can find patterns in your data and kind of see if there are any triggers that led to certain events.<br>(Diabetes)
Easy to use when commuting / on daily basis	X	Usable, Findable	For me, a device or an app is worth it if it just makes having diabetes less noticeable on a daily basis and easier to manage. I hate being RoboCop as well, but thankfully, with the Freestyle (sensor), it was finally like: 'ok, it's small enough, I can deal with it'. And sometimes I take breaks from the monitor because I get a little annoyed with hearing the alarms constantly. So, it's important to know that I have it there, that I can use it. [...] that kind of thing is helpful, because I don't like to follow the same schedule every day. I'm not big into routines, so being able to see how my blood sugar is reacting to something different [...] has changed my management completely. (Diabetes)
Spending too much time on phone	X	Usable, Accessible	So they might be nice, but it´s taking too much time and too much juggling with your phone. (Diabetes)
Taken over by technology	X	Desirable	Using a app intensively moves your energy and focus from whatever you are doing in a normal life to being a patient. You are driven by a machine (Diabetes)
Report on side effects	X	Usable, Valuable	You can inform your health, your health care providers about your supply, serious side effects, how you feel and so on. (Cancer)<br>-----------------------

			because it's not just that the care of the side effects, but also to programs their life around possible side effects, even the life of of the people next to them and even, you know, program their day jobs from at work. (Cancer)
Responsibility	X	Credible	It's it has been appearing and reappearing over and over again, who has designed the app. So what their user groups patients involve in the design and who carries the responsibility? That's also very important. (Cancer)<br>-----------------------<br>What happens if this information is incorrect? This information introducing to the device? Who takes responsibility for this? (Cancer)
Own platform	X	Useful, Desirable	So personally, I would like to have that [data on lifestyle] in a separate digital environment and not in my doctors environments (Cancer).
Information about financial support	X	Credible, Valuable	the possibility of, for example, the social support if you need or if you need economic support or or you might need information about the tax advantages or something like that (IBD)
Accessible for patients in rural areas	X	Accessible	I have difficulties still to get to have an internet connection or something like that that this could be a real burden for me when I have to use such an application or something like that. (IBD)
Accessible for newly diagnosed patients	X	Accessible	I think that even it could be more important to the quick reaction and the to my questions when I when I am newly diagnosed (IBD)

Supplementary Table S2: Literature on apps for PRO collection and UX studies

**1a) Selection of apps used in healthcare for collection of PROs**			
Study	App & Focus	Limitations	Commonality
Wac et al., 2015	*mQoL*: quality of life data collection	• Only technology savvy participants included<br>• Participants incentivized to use app risking low quality data	• The privacy of collected data is crucial and safe use for users must be provided<br>• Multimodal feedback to users in the form of text, auditory and visual aids
Ferreira, Kostakos & Dey, 2015	*AWARE*: tracking patients throughout life and integrating sensor data	• High level of phone storage required<br>• Needs improved security to meet healthcare regulation requirements<br>• Developed for research purposes	• Integration with sensor data<br>• Benchmarking visualization for users
Anders et al., 2024	*Enable*: tracking QoL and side-effects in breast cancer patients	• Limited generalizability due to high number of academics included in the sample as well	• Usefulness of reminders and alerts<br>• Appreciation of educational content and related articles

		as lower age average than the true population	
Debong, Mayer, & Kober, 2019	*mySugr:* support patient self-management of diabetes	• retrospective observational design used limiting sample control and quality of indicators	• Benefit from remote coaching, in-app education<br>• Easy daily use, simple access
Erlich et al., 2023	*MyHealthyGut:* self-management tool for celiac disease	• Small sample size (n=15)<br>• Only Apple users	• Need for customization<br>• Desire for food and medication tracking
**1b) UX-studies**			
Study	Focus	Limitations	Commonality
Gray et al., 2016	Implementing qualitative methods into ePRO app design for those with chronic diseases	• Extracting qualitative findings resource intensive<br>• Low number of patient participants	This works supports our findings which highlight the overarching desire for ongoing monitoring and tracking over time
Germini et al., 2022	Assess needs and test usability of a ePRO tool for those with hemophilia	• Limited generalizability	This work supports our findings for the patient preference for reminders and linkage with registries

Tsangaris et al., 2022	Develop a EPRO app for breast cancer care support	• Highly homogenous sample of white, high SES participants<br>•	This works supports our findings that patients desire in-app references to supportive works and communities
Zand et al., 2021	Assess patient experiments and satisfaction with a mobile tool for IBD	• High base level of technological expertise in patient sample	This work supports our findings for the personalization of app features and the ability to track symptoms
Richter et al., 2021	Assess the perceived usefulness and acceptance of ePROM collection for those suffering from rheumatoid arthritis	• Small, homogenous sample size	This works supports our findings that patients desire the ability to log their health status on an ongoing basis beyond the visits to the clinic
Conway et al., 2016	Assess user preferences and features for a diabetes management app	• Younger sample, 96% white, recruited online increasing probability of	This work support our finding that graphics/visual representations of the data are a key feature (highest ranked

		technological skills	feature of the study)

Supplementary **Figure S1**

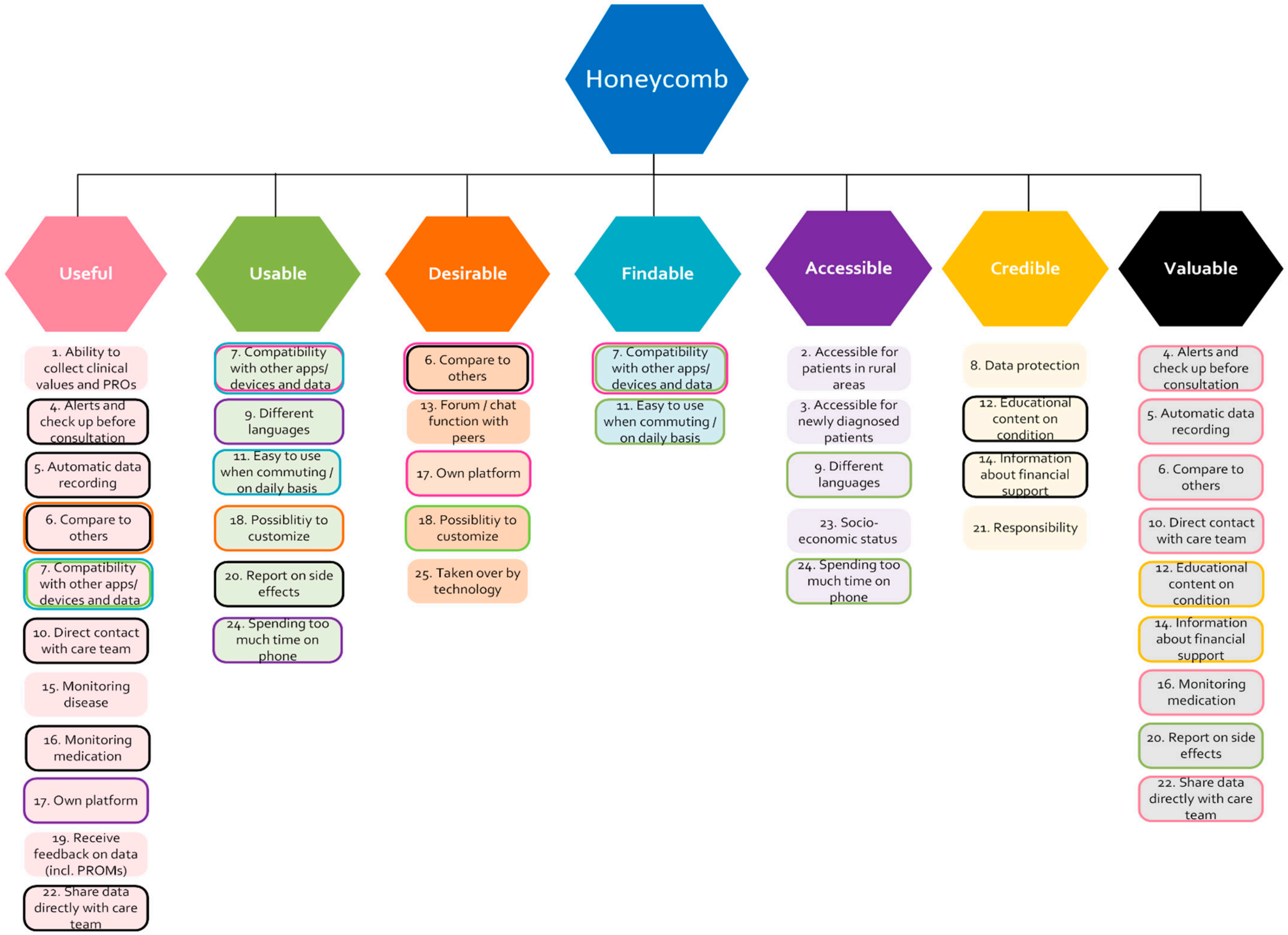

Figure S1: Themes for the focus groups mapped to the Honeycomb model

Themes are alphabetically ordered, irrespectively of perceived importance or disease groups. Themes mapped to one or more categories are presented in all categories but marked with responding outline colours.
